# Whole-transcriptome analysis reveals the interactions of mRNAs and ncRNAs to predict and validate ceRNA networks in osteosarcoma with lung metastases

**DOI:** 10.7150/jca.118620

**Published:** 2025-08-11

**Authors:** KunPeng Zhu, XingKai Wang, Lin Fan, Jiao Sun, DePing Chen, Xiaojian He, ChunLin Zhang, ChuanZhen Hu

**Affiliations:** 1 Department of Orthopedics, Shanghai Tenth People's Hospital, Tongji University, School of Medicine, Shanghai 200072, PR China.; 2 Institute of Bone Tumor, Tongji University, School of Medicine, Shanghai 200072, PR China.; 3 Department of Orthopedics, Suzhou Xiangcheng People's Hospital, Suzhou 215131, PR China.; 4 Department of Orthopedics, Huainan Chaoyang Hospital, Anhui 232007, PR China.; 5 Department of Orthopaedics, Qingpu Branch of Zhongshan Hospital, Fudan University, Shanghai 201700, PR China.

**Keywords:** osteosarcoma, lung metastasis, ceRNA, mRNA, ncRNA

## Abstract

Due to the poor prognosis and lack of effective therapy options, treating metastatic osteosarcoma (OS), particularly lung metastasis, presents significant therapeutic challenges. It has been shown that both non-coding RNAs (ncRNAs) and protein-coding mRNAs serve as essential for controlling the progression of tumors. Uncertainty persists regarding the whole expression profile and the network of regulation involving competing endogenous RNAs (ceRNAs) between mRNAs and ncRNAs in the OS lung metastasis. To fully understand variations in the expression of lncRNAs, circRNAs, miRNAs, and mRNAs, we introduced whole transcriptome sequencing (RNA-seq) in the three matched primary and lung-metastasis OS tissues used in the current study. Analysis of Kyoto Encyclopedia of Genes and Genomes (KEGG) pathways and gene ontology (GO) was carried out for mRNAs exhibiting notably distinct expression patterns. After that, the official RNA hybrid and TargetScan databases were utilized to anticipate and establish the ceRNA networks, which are made up of lncRNAs, circRNAs, miRNAs, and mRNAs. Furthermore, two created ceRNA regulatory pathways, lncRNA PCAT1/miR-370-3p/LRAT, and circ_0012586/miR-200b-5p/MFAP5, were chosen at random and verified using a variety of cell and molecular biology experiments. Ultimately, our research may reveal novel avenues for the prevention or treatment of OS lung metastasis as well as fresh evidence for the underlying mechanism.

## Introduction

The most common kind of the main malignant bone tumor in young patients is osteosarcoma (OS), which is primarily prominent in the metaphysis, such as the proximal tibia and terminal femur. 1**.** With the application of multi-drug adjuvant chemotherapy in conjunction with extensive resection surgery, the survival rate at five years has grown to 70-80 percent during the last few decades. Unfortunately, since second-line chemotherapy is not very sensitive, the survival rate at five years among individuals with numerous lung metastases dropped to fewer than 20% 2**.** To give clinicians new treatment targets and instruments, the mechanism underlying OS lung metastases needed to be further clarified 3.

Since high-throughput sequencing technology has advanced so quickly, an immense amount of non-coding RNAs (ncRNAs), once thought to be useless molecules, have been found and demonstrated to prove essential to a variety of diseases and physiological functions in cells 4. Protein-coding genes make up less than 2% of the whole genome, indicating that non-coding RNAs (ncRNAs) make up almost all of humanity's transcriptome 5. These ncRNAs may be further classified as tiny ncRNAs (less than 200 nucleotides), like miRNAs (microRNAs), lncRNAs (long ncRNAs) (more than 200 bp in length), and circRNAs (circular RNAs) having a single-stranded closed structure based on their sizes and shapes 5.

An increasing body of research indicates that these non-coding RNAs (ncRNAs) may regulate pre-transcriptional, transcriptional, and post-transcriptional levels of gene expression, hence contributing to the control of tumor development, including cell proliferation, migration, metastasis, and chemo-resistance 6. In fact, it has been revealed that a few ncRNAs regulate OS in lung metastases 7, 8. Nadya Koshkina et al. 9, for instance, found that miR-20a modulates the expression of FAS in OS cells and can be therapeutically addressed to inhibit lung metastases. According to research by Wang Y et al. 10, lncRNA DANCR decoys miR-1972 and miR-335-5p in OS to facilitate ROCK1-mediated proliferation and metastasis. This is achieved through its role as a competitive endogenous RNA (ceRNA). Furthermore, Liu YD et al. 11 discovered that via downregulating PTEN and EMP1, circ100284 fosters the invasion and migration of OS cells. Nevertheless, reports regarding the entire expression profile of mRNA and ncRNA in OS lung metastases are rare.

The explanation of a post-transcriptional layer of gene translation custody has been attributed to ceRNA networks, according to recent reports 12. A variety of RNA types, including lncRNAs, circRNAs, mRNAs, and pseudogenes, are included in these ceRNAs and may compete with one another for identical microRNA response elements (MREs) to simultaneously regulate 13. In light of their common bridge miRNAs, potential regulatory pathways such as circRNA-miRNA-mRNA and lncRNA-miRNA-mRNA might be established. As it turns out, there is growing evidence that ceRNA networks are essential for the development of tumors, including lung metastases 12, 14-17. For instance, Wang H, et al. 18 discovered that liver cancer metastasis is promoted by STAT3-mediated production of lncRNA HOXD-AS1 as a ceRNA, which is achieved via regulating SOX4. According to Luo Y. et al. 19, lncRNA DANCR regulates miRNA-33b, which in turn drives proliferation and metastasis in pancreatic cancer. Besides, Li WJ, et al. 20 demonstrated that the miR-424-5p/HMGA2 axis is the mechanism by which lncRNA LINC00355 stimulates bladder cancer cells'EMT and metastasis. Till now, there has been no study on the whole expression profile of ncRNAs and mRNA, together with the underlying ceRNA regulation networks, in the lung metastasis of OS.

In order to thoroughly recognize variations in mRNAs, lncRNAs, miRNAs, and circRNAs in the three matched primary and lung-metastatic OS tissues, we performed whole transcript genome sequencing (RNA-seq) utilizing Illumina HiSeq2000 in the present work. Gene Ontology (GO) and Kyoto Encyclopedia of Genes and Genomes (KEGG) pathway analyses were employed to analyze the function of mRNAs mediated via lncRNA-miRNA or circRNA-miRNA interactions with significant variations in the expression of OS with lung metastases. The authoritative TargetScan and RNA hybrid databases were subsequently utilized to forecast and build the ceRNA networks of mRNAs, lncRNAs, miRNAs, and circRNAs. Finally, two built ceRNA modulatory pathways were screened and verified using a series of cellular and molecular biology assays. Our research may offer fresh insight into the underlying mechanisms of ceRNA networks in OS lung metastases, rupture up novel possibilities for the acquisition of preventative or therapeutic targets.

## Methods

### Cell lines culture

The MG63 and 143B cells (human bone cell lines) were purchased from the American Type Culture Collection. The cell lines were then maintained at 37°C in a humidified CO_2_ (5%) environment using DMEM mixed with 10% fetal bovine serum (Gibco, Gran Island, NY, USA), 100 mg/mL of streptomycin (Invitrogen), and 100 U/mL of penicillin. In Ham's F12/DMEM mixed with 10% FBS, 100 U/mL penicillin, and 100 mg/mL streptomycin, ordinary osteoblast cell lines (hFOB1.19) were grown. These cells were acquired from the Chinese Cell Bank of the Chinese Academy of Sciences (Shanghai, China).

### Human tissue samples

This study comprised 40 primary osteosarcoma patients who, at Shanghai Tenth People's Hospital, received full surgical excision of both the original and lung metastatic lesions between 2013 and 2020. All patients gave their written informed permission, and the Shanghai Tenth People's Hospital Ethics Committee allowed the study. The same pathologist examined the slides from each patient to confirm the diagnosis. Each resected specimen was put into liquid nitrogen right away and kept at -80°C for storage.

### RNA extraction and quality control

Following the company's instructions, the entire RNA was extracted from cells as well as tissues via a TRIzol reagent (Life Technologies, CA, USA). With the assistance of the NanoDrop ND-1000 (Wilmington, DE, USA), the amount and purity of the entire RNA from the samples were measured. The Agilent 2100 Bioanalyzer (Agilent Technologies, Santa Clara, CA, USA) was utilized to assess RNA integrity. The samples that had an RNA Integrity Number (RIN) of no less than seven underwent the following study.

### Creating a small RNA sequencing library

Three micrograms of entire RNA per sample ended up serving as the input element for the small RNA library. The NEBNextR Multiplex Small RNA Library Prep Set for Illumina (NEB, USA) was employed to produce sequencing libraries in conformity with the manufacturer's guidelines. After that, each sample's sequences were identified by adding the index codes to it. In short, the 3' end of miRNA was selectively and directly ligated to the NEB 3' SR Adaptor. Following the 3' ligation process, the excess 3' SR Adaptor that was left free after the 3' ligation reaction hybridized with the SR RT Primer, converting the single-stranded DNA adaptor into the double-stranded DNA monomer. Preventing the creation of adaptor dimers requires taking this crucial step. Furthermore, in the subsequent ligation step, dsDNAs do not ligate to the 5' SR Adaptor as they do not serve as candidates for ligation mediated by T4 RNA Ligase 1. Subsequently, the 5' ends of the miRNAs were ligated onto the adaptor. M-MuLV Reverse Transcriptase was implemented for generating first-strand cDNA (RNase H-). PCR amplification was performed using LongAmp Taq 2X Master Mix, SR Primer for Illumina, and index (X) primer. On an eight percent polyacrylamide gel, the PCR products were purified at 100 V for 80 minutes. A mixture of the 3' and 5' adaptors and short noncoding RNA measuring 140-160 bp was employed to disintegrate and extract segments of DNA in the 8-μL elution solution. Lastly, we applied the DNA High Sensitivity Chips on the Agilent Bioanalyzer 2100 machine to evaluate the quality of the libraries.

### Building cDNA libraries along with sequencing with high throughput

Using Trizol (Invitrogen), the entire RNA was extracted from both categories under the supplier's instructions. Strand-specific cDNA libraries were created in accordance with previously described procedures, and an Illumina HiSeq2000 sequencer (Jian-Wen biotech, Wuhan, China) was applied in a paired-end run with a one hundred bp read length to sequence the libraries. Clean reads were adopted throughout the ensuing analyses. Clean paired-end reads were matched to the reference genome using TopHat (version 2.0.6). Cufflinks (version 2.0) served for constructing each sample's transcriptome. Gene or lncRNA (circRNA) expression levels were measured using reads per kilo base of model per million base pairs sequenced (RPKM), whereas miRNA expression levels were ascertained using transcript per million (TPM). The DEGseq system was implemented to figure out the distinctions within the various groups.

### Enrichment analysis

A functioning study known as GO analysis links GO categories to mRNAs that exhibit differential expression. The source from which the GO categories are derived is Gene Ontology (www.geneontology.org), which is composed of three organized frameworks of specified terms describing the properties of genetic products. Pathway analysis was done for mRNAs that expressed differently utilizing the most recent version of the KEGG (Kyoto Encyclopedia of Genes and Genomes, http://www.genome.jp/kegg) database. This made it possible for us to determine the process in biology that the considerably enriched mRNAs participated in.

### Outline the process of ceRNA network recognition

For the calculation of Pearson's correlation coefficient and *p*-value corresponding to the miRNA target, the corresponding expression levels of mRNAs, miRNAs, and lncRNAs or circRNAs were taken into consideration. A *p*-value below 0.05 and Pearson's correlation coefficient of more than 0.8 indicated negatively correlated couples, which were chosen for additional examination. For miRNA-lncRNA/circRNA/mRNA and mRNA-lncRNA/circRNA, these were the anticipated couples. Further analysis was then conducted using common pairings based on the forecast pairs obtained from attaching regions and the forecast pairs earned via the transcript levels of mRNA, lncRNA (circRNA), and miRNA. The ceRNA score was predicted using shared pairings of miRNA-Mrna/lncRNA/circRNA, by the ceRNA prediction principle, as expressed in the following formula.



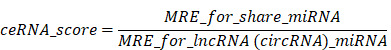



Then, actual ceRNAs were found to be the common pairs via the ceRNA score principle and the anticipated lncRNA (circRNA)-mRNA pairs following the levels of lncRNA (circRNA) and mRNA (Pearson's correlation coefficient). Lastly, we employed Cytoscape software (V.3.2.1) to construct circRNA/lncRNA/miRNA inter-crosslinked ceRNA networks based on the available co-expression data.

### Western blot (WB)

Osteosarcoma cells' total proteins were collected and subsequently put into 10% sodium dodecyl sulfate-polyacrylamide gel electrophoresis (SDS-PAGE). Following protein separation, the membranes made of polyvinylidene difluoride (PVDF) were blocked for one hour with 5% nonfat milk. PVDF membranes were then incubated overnight at 4°C (Santa Cruz, 1:1000 dilution), comprising anti-LRAT and anti-MFAP5 under primary antibodies. The secondary antibody (1:500 goat anti-rabbit IgG) was then incubated for one hour. The expression of the protein was measured using a chemical luminescence monitoring kit (Amersham Pharmacia Biotech) and compared to β-actin as the intrinsic control.

### Quantitative real-time PCR (qRT-PCR)

Osteosarcoma cells as well as tissues received treatment with the TRIzol reagent (Invitrogen) to extract total RNA. Next, 200 ng of isolated entire RNA was converted to cDNA through the PrimeScriptRT reagent Kit (Takara Bio Company, Shiga, Japan). ABI PRISM 7500 Sequence Detection System (Applied Biosystems, Foster City, CA, USA) was set up to carry out qRT-PCR on this using a SYBR Green Kit (Takara Bio Company), with the “housekeeping” gene GAPDH acting as a negative control. The relative quantification of gene expression levels was ascertained by means of the 2^-ΔΔCt^ approach. All the primers were synthesized by Sangon (Shanghai, China). **Table 1** showcases the primers.

### Cell transfection

GenePharma (Shanghai, China) developed and fabricated small inter (si) RNAs targeting lncRNA PCAT1 (or circ_0012586) and a matched reference control (si-nc), along with mimics and inhibitors of miR-370-3p or miR-200b-5p. Following the manufacturer's instructions, 143B (or MG63.2) cells were transfected leveraging Lipofectamine 2000 (Invitrogen). **Table 2** lists the designed sequences.

### CCK-8 assay

Cell viability was assessed with the Cell Counting Kit-8 (CCK-8, Dojindo, Japan) for 48 hours after transfection in 96-well plates, per the company's directions. The absorbance of the wells at 450 nm was quantitatively determined for individual wells by using an enzyme labeling apparatus (Biotek, Winooski, Vermont, USA). All the above studies were carried out at least three times.

### Transwell assays

The transfected cells were inoculated into six-well plates for co-culture and incubated the whole night. After scraping the cell layer using a sterile tip on a pipette, the layer was cleaned with a medium. For 48 hours, the cells were grown in a medium with 1% FBS. Twenty-four-well transwell chambers were utilized in the invasion experiments, and the upper chambers were covered with Matrigel (BD Bioscience). The top compartment was seeded with 1.0×10^5^ cells in 100 uL serum-free DMEM media, while the lower chamber was filled with 500 uL medium containing 10% FBS. Following a 48-hour incubation period, cotton swabs were employed to eliminate all cells on the membrane's surface, and those that crossed it were tallied and stained with crystal violet.

### Luciferase gene reporter assay

After plating 293T cells with a concentration of 5×10^4^ cells/well within the 96-well plates, they were transfected. Using PCR amplification, the 3'-UTR of the lncRNAs PCAT1 cDNA and LRAT were increased. Subcloning the resultant clones into the luciferase reporter vector pGL3-basic (Promega, Madison, WI, USA) was the next step. Renilla Luciferase served as the reporter in control. Following the manufacturer's instructions, Lipofectamine 2000 (Invitrogen, California, USA) was employed to transfect miR-370-3p mimics or negative controls (100 nM). After lysing the cells, the activity of luciferase was measured. We normalized these values using the Dual-Luciferase Reporter Gene Assay Kit (Promega) as a Renilla luciferase transfection control. This approach was also applied to verify the connection across circ_0012586 and miR-200b-5p or miR-200b-5p and MFAP5.

### Statistical analyses

In a statistical study, the student's t-test was utilized for contrasting the two factors found in the RNA sequencing data. According to sequencing of RNA analysis, a change with folding (FC) of over 2 or not larger than 0.5 with a *p*-value less than 0.05 is deemed statistically significant. The Benjamini-Hochberg was performed to adjust the *p*-value on RNA sequencing analysis. The qRT-PCR data were processed using the 2^-ΔΔCt^ technique, and the expressed amount of each mRNA, miRNA, lncRNA, and circRNA was denoted by FC. The *p*-value less than 0.05 was considered statistically significant.

## Results

### Expression profiles of mRNAs, lncRNAs, miRNAs and circRNAs

**Figure 1:**
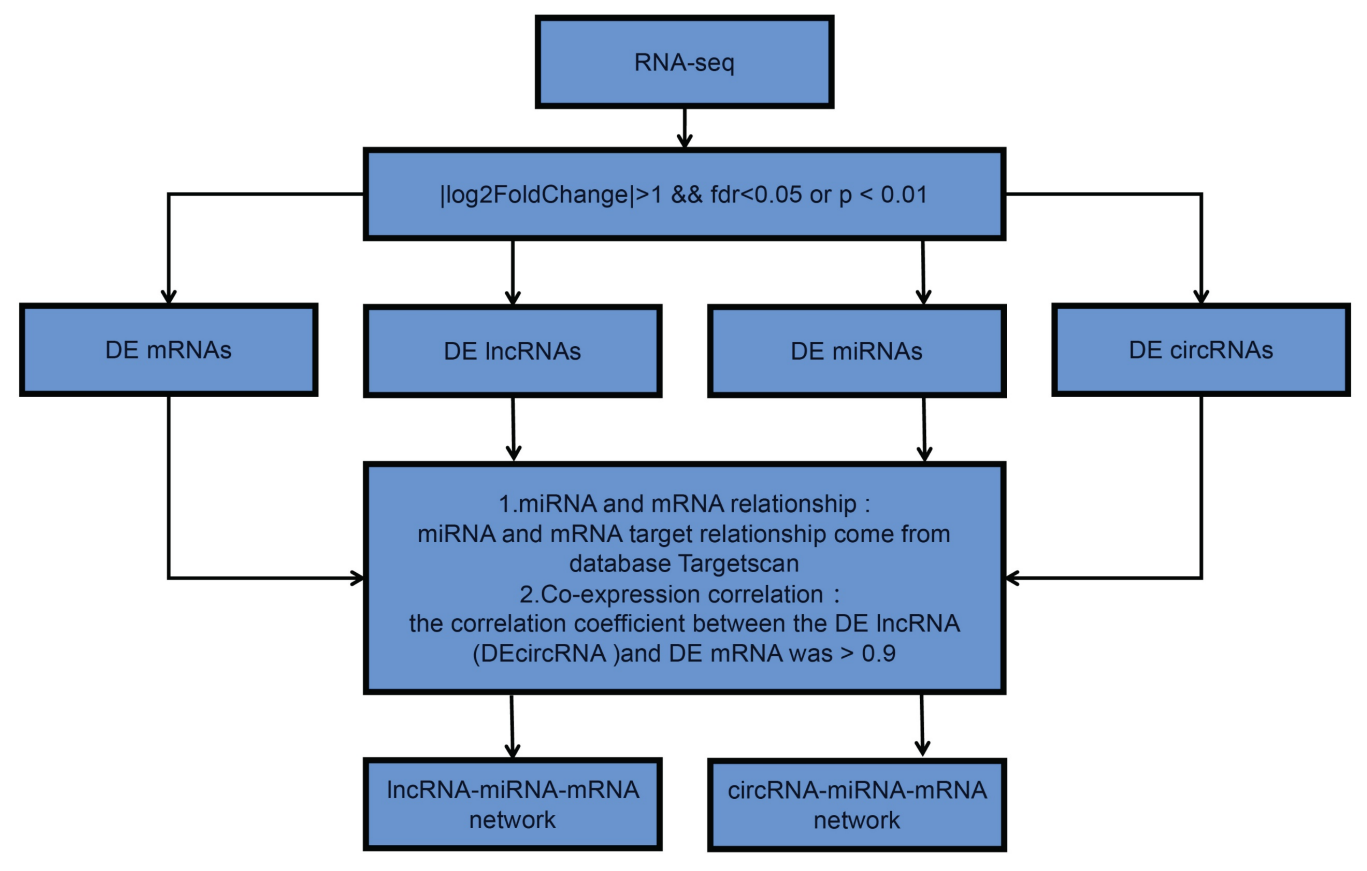
**Flow chart of this study design.** DE is short for “differently expressed”.

**Figure 2:**
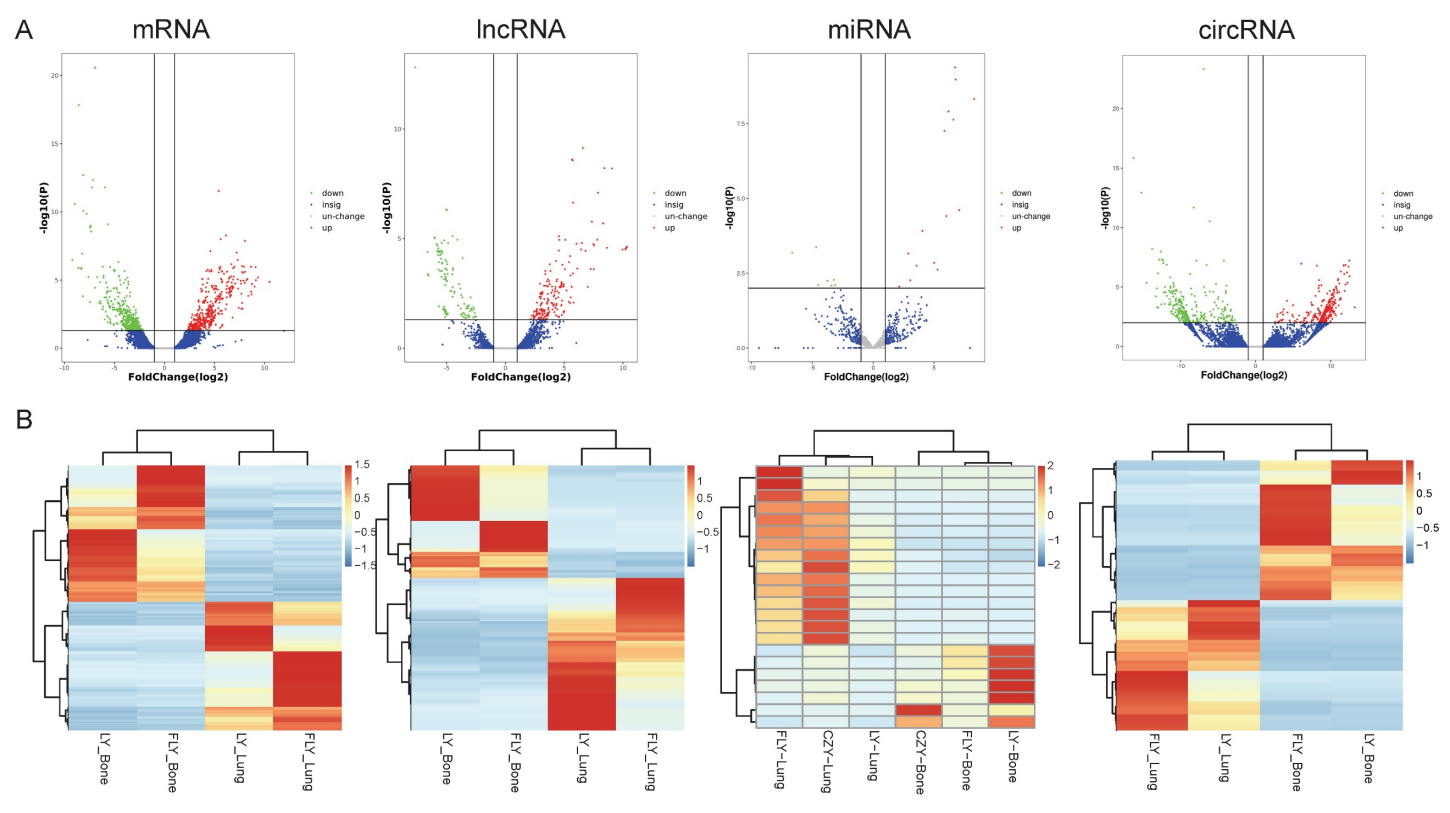
**Expression profiles of lncRNAs, circRNAs, miRNAs, and mRNAs.** DE ncRNAs and mRNAs in the three paired primary and lung-metastasis OS tissues were shown using volcano plots **(A)** and heatmap** (B)**.

Utilizing the Illumina Hiseq2500 platform, we obtained the transcriptome sequencing data (lncRNA, circRNA, miRNA, and mRNA) from primary and lung-metastatic OS groups. Then, we identified differently expressed (DE) RNAs (lncRNAs, circRNAs, miRNAs, mRNAs) with the thresholds of FC > 2 or ≤ 0.5 along with* p*-value < 0.05 and FDR < 0.05 from three primary and lung-metastatic OS tissues. **Figure 1** demonstrates the present study's methodology and analytical approach. **Figure 2** displays the variations in DE ncRNA and mRNA expression in several samples. In comparison to the primary OS tissues, the lung-metastatic OS tissues contained 159 up- and 216 down-regulated DE lncRNAs (375 in total), 323 up- and 345 down-regulated DE circRNAs (667 in total),7 up- and 15 down-regulated DE miRNAs (22 in total), and 593 up- and 626 down-regulated DE mRNAs (1219 in total). As shown in **Table 3**, CFAP47 was the most up-regulated mRNA with 42.5 FC, whereas AC132217.2 was the most down-regulated mRNA with 123.6 FC. AC010197.1 (6.59 FC), circ_0077 054 (12.47 FC), and miR-375-3p (8.30 FC) were the significantly up-regulated lncRNA, circRNA, and miRNA, respectively. In addition, the most down-regulated lncRNA, circRNA, and miRNA were AC132217.1 (-7.68 FC), circ_0020792 (-6.91 FC), and miR-483-3p (-6.65 FC), respectively.

### Enrichment analysis of these DE RNAs

**Figure 3:**
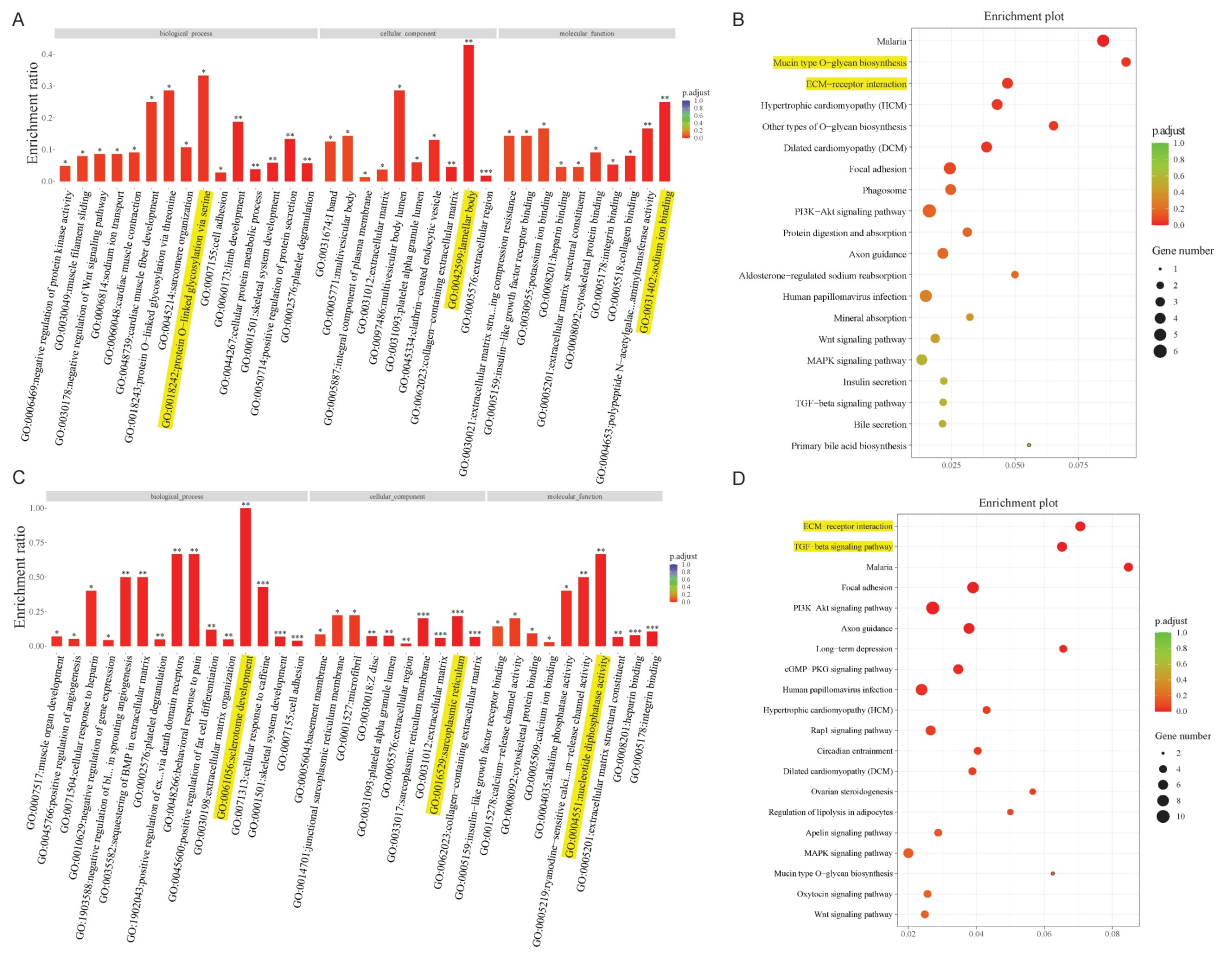
**Bioinformatics analysis in the ceRNA network.** GO and KEGG pathway analysis for the mRNAs regulated by the lncRNA-miRNA network **(A, B)** and circRNA-miRNA network** (C, D)**.

Taking the above analysis as a basis, we constructed two lncRNA-miRNA-mRNA/circRNA-miRNA-mRNA networks containing lncRNA, circRNA, miRNA, and mRNA. Given the complexity of the networks, we extracted the mRNAs associated with the two networks, for subsequent analysis. GO and KEGG enrichment analysis are commonly used to analyze mRNA function and the involved pathways. DE mRNAs are mostly implicated in protein O-linked glycosylation via serine (biological process), lamellar body (cellular component), and sodium ion binding (molecular function) within the lncRNA-miRNA-mRNA network, according to GO analysis **(Figure 3A)**. Concurrently, KEGG pathway enrichment analysis revealed that these mRNAs mostly functioned in relation to malaria, O-glycan biosynthesis of the mucin type, and other forms of O-glycan biosynthesis **(Figure 3B)**. The findings of GO analysis of mRNAs in the circRNA network, which included sclerotome development (biological process), sarcoplasmic reticulum (cellular component), and nucleotide diphosphatase activity (molecular function), were displayed in **Figure 3C**. Additionally, pathway enrichment analysis showed that these mRNAs are connected to activities related to malaria, the TGF-beta signaling pathway, and the ECM-receptor interaction **(Figure 3D)**.

### Bioinformatics for constructing the ceRNA network

**Figure 4:**
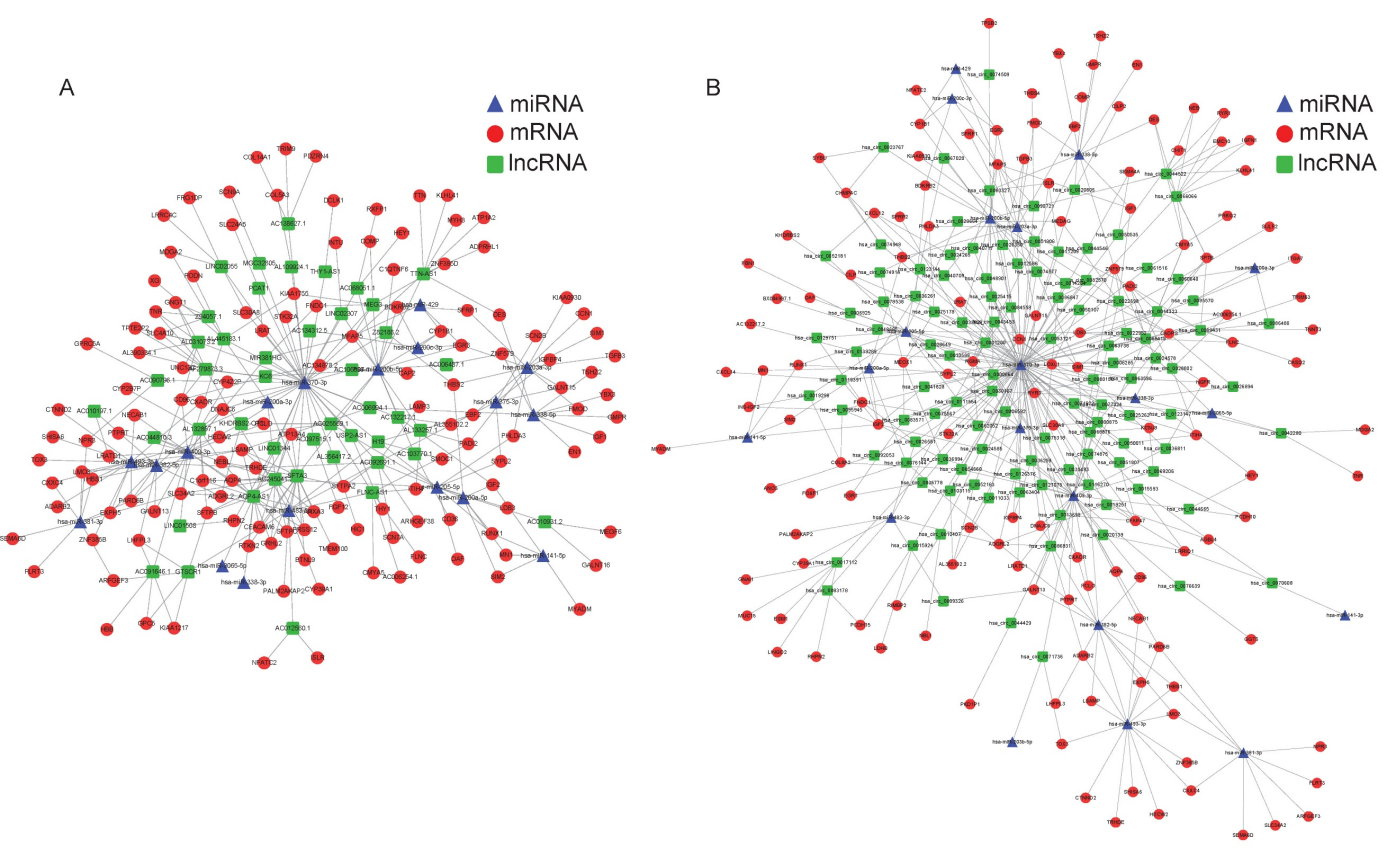
**Constructed ceRNA networks based on the differently expressed miRNAs.** LncRNA-miRNA-mRNA network** (A)** and circRNA-miRNA-mRNA network **(B)** were shown respectively. The red nodes represented mRNAs, green nodes for lncRNAs or circRNAs, blue frames for miRNAs, and the edges represented the competing interactions among them.

As far as we know, the regulation mechanism of ceRNA serves a critical role in the interaction between mRNA and non-coding RNAs, such as circRNA, lncRNA, and miRNA. Since there were the fewest differentiating expressions of miRNAs, we attempted to build the regulatory network at the center of miRNAs. Using the TargetScan database, we initially determined the target mRNAs of these miRNAs and conducted a thorough comparison with DE mRNAs based on sequencing results. Then the lncRNAs (circRNAs) which possess the potential binding sites with these DE miRNAs were predicted by the RNAhybrid and further screened by the mutually negative regulatory relationship in our RNA-seq results. Thus, we constructed an interaction network of lncRNA/cirRNA cross-linked with miRNA and mRNA. A total of 45 lncRNAs, 18 miRNAs, and 102 mRNAs were employed to create the 538 lncRNA-miRNA-mRNA pathways **(Figure 4A)**. In addition, 818 cirRNA-miRNA-mRNA pathways involving 102 circRNAs, 20 miRNAs, and 125 mRNAs were established **(Figure 4B)**. Given the intricacy of the interactions between ncRNAs and mRNAs, these ceRNA regulatory linkages might be significant in the process of OS lung metastasis.

### Random selection and validation of LncRNA PCAT1/miR-370-3p/LRAT pathway

**Figure 5:**
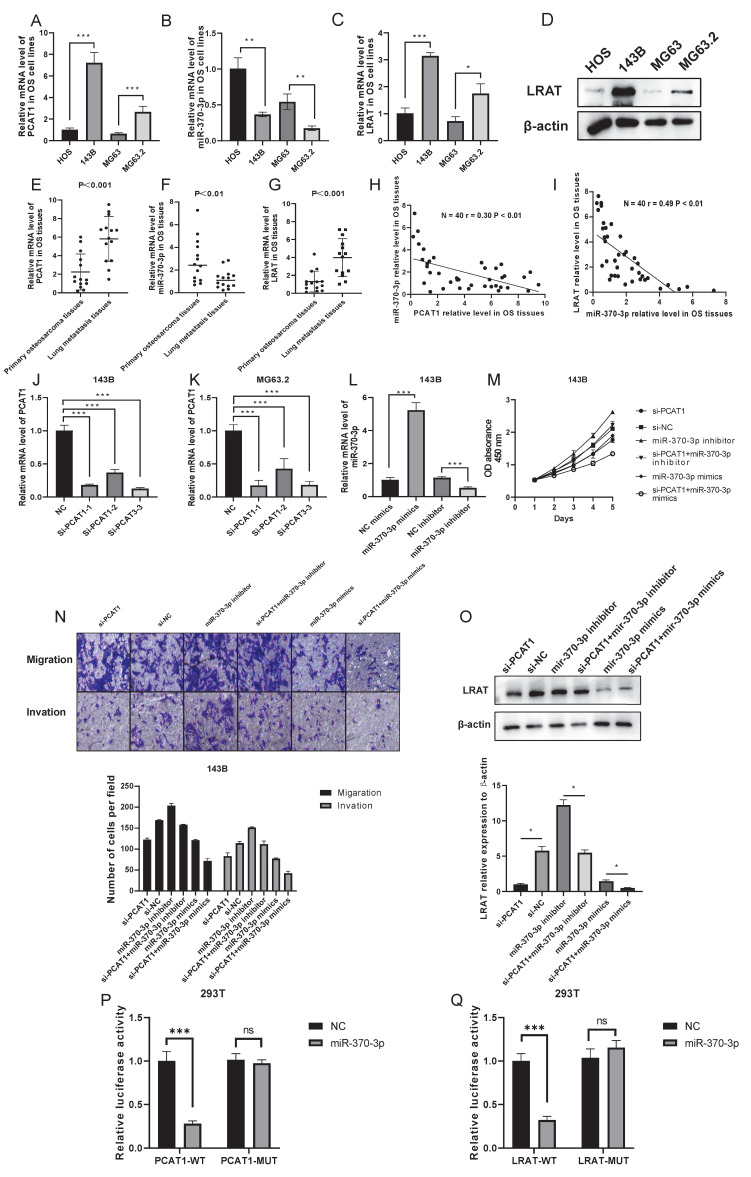
** LncRNA PCAT1/miR-370-3p/LRAT pathway was randomly chosen to verify the feasibility of the constructed lncRNA-miRNA-mRNA network.** The mRNA expression levels of PCAT1** (A)**, miR-370-3p** (B)**, and LRAT **(C)** were examined by the qRT-PCR in highly metastatic OS cells and the control. The protein expression level of LRAT **(D)** was examined by the WB in highly metastatic OS cells and the control. The mRNA expression levels of PCAT1** (E)**, miR-370-3p** (F)**, and LRAT **(G)** were examined by the qRT-PCR in the paired primary and lung-metastasis OS tissues. **(H-I)** The correlation between PCAT1 and miR-370-3p as well as miR-370-3p and LRAT in OS tissues. **(J-K)** The qRT-PCR analysis of the effect on knockdown of PCAT1 expression by siRNA in the 143B and MG63.2 cell lines. **(L)** The qRT-PCR analysis of the effect on mimics or inhibitors of miR-370-3p in the 143B cells. **(M-N)** Cell viability, migration, and invasion of OS cells were regulated by lncRNA PCAT1 and miR-370-3p. **(O)** LRAT expression was regulated by the lncRNA PCAT1 and miR-370-3p.** (P)** Luciferase activity assay showed that PCAT1 could combine with miR-370-3p.** (Q)** Luciferase activity assay showed that miR-370-3p could combine with 3'UTR of LRAT mRNA.

We arbitrarily chose lncRNA PCAT1/miR-370-3p/LRAT as the verified route in order to highlight the noteworthy importance of the ceRNA network. As shown in **Figure 5A-G**, lncRNAs PCAT1 and LRAT showed high expression in OS cells and tissues. Their expression was far more enhanced in lung metastases compared to primary osteosarcoma tissues. MiR-370-3p, however, possessed the opposite trend. We further found that the expression level of miR-370-3p was negative with the level of lncRNA PCAT1 and LRAT mRNA in the OS tissues (N=40), which indicated the potential regulatory relationship among them **(Figure 5H-I)**. Subsequently, we synthesized siRNAs against the lncRNA RNA PCAT1 and introduced them into the 143B (MG63.2) cell line to investigate their possible function in lung metastasis. Utilizing qRT-PCR to examine the transfection impact, si-PCAT1-3 (termed si-PCAT1) showed the most effective inhibition and was employed for further investigation **(Figure 5J-K)**. Meanwhile, in 143B cells, we evaluated the effect of miR-370-3p mimics and inhibitors **(Figure 5L)**. The results showed that the cell viability, migration, and invasion were decreased by the si-PCAT1 transfection which might be recovered by transfecting with miR-370-3p inhibitor or aggravated by transferring with miR-370-3p mimics **(Figure 5M-N)**. Furthermore, the application of si-PCAT1 resulted in a considerable drop in the level of LRAT protein expression in 143B cells; however, the addition of miR-370-3p inhibitor was able to restore its expression **(Figure 5O)**.

Further evidence that miR-370-3p might target binding to the 3' non-coding region (3'-UTR) of lncRNA PCAT1 and LRAT mRNAs with complementary binding sites in this area was provided by a bioinformatics study. We created luciferase reporter plasmids that carried both mutant-type (MUT) and wild-type (WT) PCAT1 or LRAT. It is clear that there was a decrease in reporter activity in 293T cells when the luciferase reporter plasmid carrying the WT-PCAT1 was co-transfected with miR-370-3p mimics **(Figure 5P)**. Luciferase reporter gene assay also further strengthened the existence of intermolecular interaction between miR-370-3p and LRAT **(Figure 5Q)**. In conclusion, a potential effect of molecular interactions in lncRNA PCAT1/miR-370-3p/LRAT pathway for OS lung metastasis was uncovered by the above results.

### Random selection and validation of Circ_0012586/miR-200b-5p/MFAP5 pathway

**Figure 6:**
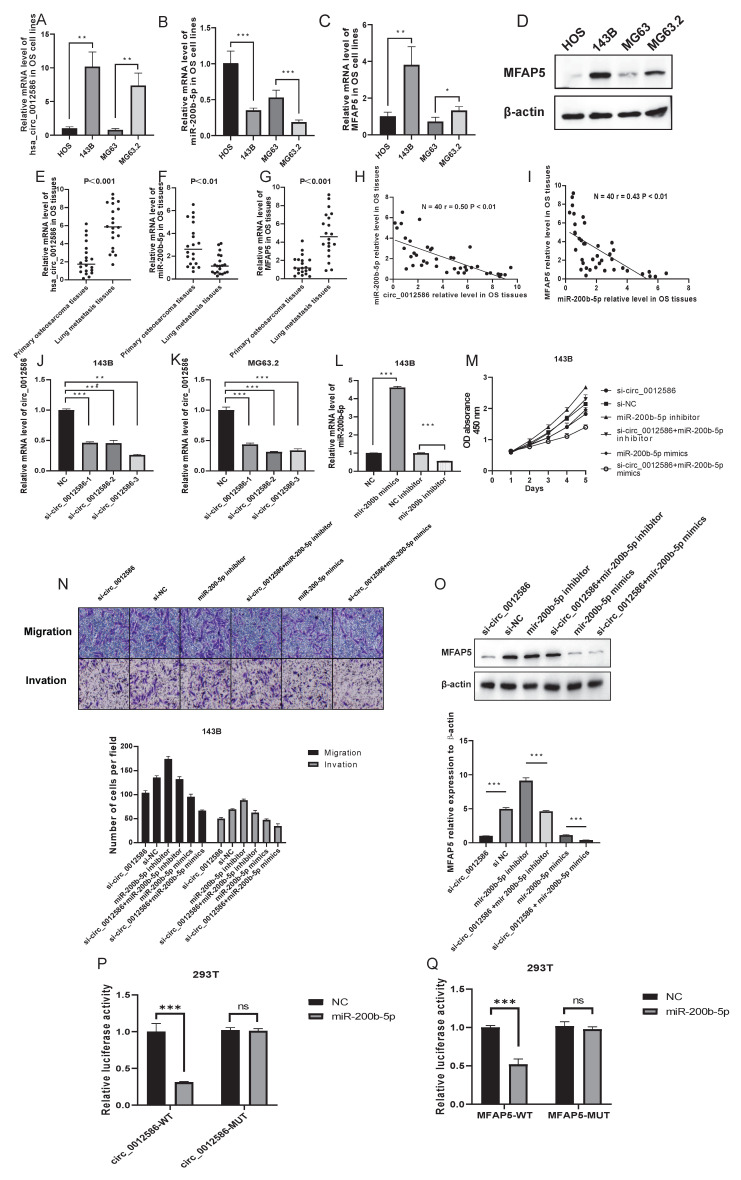
** Circ_0012586/miR-200b-5p/MFAP5 pathway was randomly chosen to verify the feasibility of the constructed circRNA-miRNA-mRNA network.** The mRNA expression levels of circ_0012586 **(A)**, miR-200b-5p **(B)**, and MFAP5** (C)** were examined by the qRT-PCR in highly metastatic OS cells and the controlled. The protein expression level of MFAP5 **(D)** was examined by the WB in highly metastatic OS cells and the control. The mRNA expression levels of circ_0012586** (E)**, miR- 200b-5p **(F)**, and MFAP5** (G)** were examined by the qRT-PCR in the paired primary and lung-metastasis OS tissues. **(H-I)** The correlation between circ_0012586 and miR-200b-5p as well as miR-200b-5p and MFAP5 in OS tissues was shown. **(J-K)** The qRT-PCR analysis of the effect on knockdown of circ_0012586 expression by siRNA in the 143B and MG63.2 cell lines was shown. **(L)** The qRT-PCR analysis of the effect on mimics or inhibitors of miR-200b-5p in the 143B cells. **(M-N)** Cell viability, migration, and invasion of OS cells were regulated by circ_0012586 and miR-200b-5p. **(O)** LRAT expression was regulated by the circ_0012586 and miR-200b-5p.** (P)** Luciferase activity assay showed that circ_0012586 could combine with miR-200b-5p in the 293T cells. **(Q)** Luciferase activity assay showed that miR-200b-5p could combine with 3'UTR of MFAP5 mRNA in the 293T cells.

Similarly, the anticipated circ_0012586/miR-200b-5p/MFAP5 pathway was selected at random to assess the noteworthy importance of the existing network. We first detected the expression of the three molecules of the network in cells and tissues, and the results were consistent with the sequencing data. Interestingly, we found that the expression trends of circ_0012586, miR-200b-5p, and MFAP5 in OS cells and tissues, as well as OS lung metastatic tissues, were consistent with the lncRNA PCAT1/miR-370-3p/LRAT pathway **(Figure 6A-G)**. Additionally, we discovered that in the OS tissues (N=40), the expression level of miR-200b-5p was negative with circ_0012586 and MFAP5 mRNA expression level, suggesting the potential regulatory relationship among them **(Figure 6H-I)**. We selected three candidate siRNAs targeting circ_0001258, among which si-circ_0001258-3 had the highest transfection efficiency, demonstrated the most excellent inhibition effect, and was utilized for subsequent analysis **(Figure 6J-K)**. Meanwhile, in 143B cells, we evaluated the effect of miR-200b-5p mimics and inhibitors **(Figure 6L)**. We found that the cell viability, migration, and invasion of 143B cells were decreased by the si-circ_0001258 transfection which could be rescued by transfecting with miR-200b-5p inhibitor or aggravated by transfecting with miR-200b-5p mimics **(Figure 6M-N)**. Furthermore, down-regulating the circ_0001258 by siRNA resulted in a substantial drop in MFAP5 protein expression, which could be reversed by suppressing the expression of miR-200b-5p in the 143B cells **(Figure 6O)**.

Moreover, bioinformatics analysis further demonstrated that miR-200b-5p holds the same complementary binding sites with 3'-UTR of circ_0001258 and MFAP5 mRNA, which were also demonstrated by the luciferase reporter gene assay **(Figure 6P-Q)**. Overall, we constructed the circ_0012586/miR-200b-5p/MFAP5 pathway with a potential implication in OS lung metastasis.

## Discussion

Lung metastasis is still a tricky clinical problem in OS treatment owing to a lack of effective prevention and treatment strategies 21. It often means a poor prognosis for OS patients who have lung metastasis and it is very urgent to further clarify its underlying mechanism to uncover some novel treatment targets 22-24. During the past few decades, more and more ncRNAs, such as miRNA, lncRNA, and circRNA, were found and reported to play essential roles in cancer metastasis 15-17, 25.

As is well known, mRNAs exhibit diverse biological functions through the regulation of ncRNAs, and the ceRNA network contributes to unraveling the complex regulatory relationships among these RNAs 12, 13. Currently, the potential role of the ceRNA regulatory network on cancers, including OS, has been reported. For example, Bioinformatics studies showed that a ceRNA network based on lncRNAs (AL162511.1, AC090673.1, and AL365259.1) could serve as a potential biomarker for the diagnosis and treatment of thyroid cancer 26. In the opinion of Yang et al., who developed a lncRNA-based predictive model for hepatocellular carcinoma, seven lncRNAs may be effective as prognostic indicators for the disease 27. The therapy of tongue squamous carcinoma is aided by the discovery discovered by Zhou RS et al. on the molecular regulatory function of the lncRNA-centered ceRNA network in this disease 28. Simultaneously, the outcomes of survival and differential studies suggested that the ceRNA network has a regulatory role in melanoma, influencing the disease's advancement 29. The ceRNAs also have a crucial regulatory role in osteosarcoma 30. Additionally, we demonstrated in our earlier study that the ceRNA network may be a viable target for osteosarcoma chemoresistance therapy 31. Nevertheless, reports regarding the thorough transcriptional profiles and analysis of the mRNAs and ncRNAs implicated in OS lung metastasis based on the deep analysis of the RNA sequencing in the tissues are rare. In this work, we utilized whole transcriptome RNA sequencing to concurrently determine the lncRNA, circRNA, miRNA, and mRNA gene expression profile in the three matched primary and lung-metastasis OS tissues. DE ncRNAs and mRNAs were found to include 375 lncRNAs, 667 circRNAs, 22 miRNAs, and 1219 mRNAs that fulfilled the screening requirements (FC > 2 or ≤ 0.5, and *p*-value < 0.05). Enrichment analysis revealed that DE mRNAs were mainly enriched in O-glycan biosynthesis, ECM-receptor interaction, and TGF-beta signaling pathway.

Among the regulatory mechanisms associated with cancer metastasis, previous studies have revealed that lncRNAs and circRNAs can function as ceRNAs to impact metastatic development by sponging miRNAs to regulate mRNAs. For example, He SL, et al. 32 the regulation of lncRNA KCNQ1OT1 and mRNA EIF2B5 has a significant impact on the metastatic development of ovarian cancer. The circRNA cRAPGEF5/miR-27a-3p/TXNIP axis successfully prevented renal cell cancer metastases, according to mechanistic research 33. Furthermore, ncRNAs such circRNA_0000140 34, exosomal lncRNA PCAT1 35, lncRNA ODRUL 36, and circular RNA PVT1 37 have the ability to influence the metastasis of cancer and may function as biological markers for diagnostic and prognostic purposes. We systematically investigated the possible role of ncRNAs in OS lung metastasis throughout our work. The ceRNA regulation networks focusing on lncRNA/circRNA and linked to OS lung metastasis were screened and integrated using bioinformatics analysis. We constructed the ceRNA regulatory networks including 538 lncRNA-miRNA-mRNA pathways (45 lncRNAs, 18 miRNAs, and 102 mRNAs) and 818 cirRNA-miRNA-mRNA pathways (102 circRNAs, 20 miRNAs, and 125 mRNAs). Two estimated pathways, lncRNA PCAT1/miR-370-3p/LRAT, and circ_0012586/miR-200b-5p/MFAP5, were further chosen and validated by a series of molecular biology and cell experiments in order to strengthen the validity of our findings.

The functions of PCAT1, miR-370-3p, and LRAT in OS lung metastasis or cancer chemo-resistance, however, are not well documented. Although prior research has demonstrated that the lncRNA PCAT-1 stimulates OS development, its impact on metastasis has not been investigated 38. Similarly, LRAT and miR-370-3p can function as important regulatory molecules in the ceRNA regulatory network to affect the development of various tumor types 39-41. However, there have been no reports between LRAT and OS as well as the lncRNA PCAT1/miR-370-3p/LRAT pathway in cancer. We have shown that in highly metastatic OS cell lines and tissues, lncRNAs PCAT1 and LRAT are considerably overexpressed relative to controls, whereas miR-370-3p exhibits a declining trend. The results of phenotypic assays showed that lncRNA PCAT1 may upregulate LRAT to facilitate OS cell invasion, migration, and proliferation; however, this effect was blocked when the expression of miR-370-3p increased. Mechanistically, by sponging miR-370-3p, lncRNA PCAT1 as a ceRNA increased the expression of LRAT. Thus, the lncRNA PCAT1/miR-370-3p/LRAT pathway influencing OS lung metastasis was created and verified in the current study.

As for the circ_0012586/miR-200b-5p/MFAP5 pathway, there was still no report about circ_0012586 and cancer. However, some reports have demonstrated the role of MFAP5 in the progression of breast cancer 42, bladder cancer 43, colon cancer 44, and so on^45^. The multiple contribution of circ_0012586 and MFAP5 in OS lung metastasis was discovered for the first time in this present study. We have demonstrated that in highly metastatic OS cell lines and tissues, circ_0012586 as well as MFAP5 are considerably overexpressed relative to controls, whereas miR-200b-5p exhibits a declining trend. Similar in function to the lncRNA PCAT1/miR-370-3p/LRAT pathway, circ_0012586 sponging miR-200b-5p regulates MFAP5, which in turn impacts OS lung metastasis.

### Limitations and future directions

This study has several limitations that also inform future research directions. First, while RNA-seq analysis of three paired primary/metastatic tissues identified DE RNAs, validation in 40 clinical samples via qRT-PCR highlights the need for larger independent cohorts (e.g., TCGA-OS dataset) to confirm ceRNA network consistency. Second, although two ceRNA pathways (lncRNA PCAT1/miR-370-3p/LRAT and circ_0012586/miR-200b-5p/MFAP5) were validated, the full network comprises 538 lncRNA-miRNA-mRNA and 818 circRNA-miRNA-mRNA interactions, necessitating high-throughput screening (e.g., CRISPR-Cas9) to characterize unvalidated regulatory relationships. Third, the clinical relevance of PCAT1, circ_0012586, LRAT, and MFAP5 remains unproven in animal models, requiring orthotopic OS lung metastasis xenografts to evaluate their roles in metastatic progression.

To address these, future studies will: expand ceRNA network validation in larger patient cohorts and independent datasets to identify robust metastatic biomarkers; use functional genomics (e.g., shRNA screens) to characterize unvalidated pathways in OS cell migration/invasion; investigate in vivo efficacy of targeting PCAT1 or circ_0012586 via xenografts to bridge translational gaps. These efforts will strengthen clinical relevance and facilitate ceRNA-based therapeutic strategies for OS lung metastasis.

## Conclusions

We performed a whole transcriptome analysis using primary and lung metastatic osteosarcoma samples and obtained DE ncRNAs and mRNAs. Moreover, enrichment analysis facilitated in-depth exploration of the potential functions of these DE RNAs. The ceRNA network we constructed centered on lncRNA/circRNA is an easier way for us to understand the mechanism of osteosarcoma lung metastasis. This study is the first to investigate the variation of mRNA and ncRNA expression profiles in OS lung metastases based on transcriptome sequencing in tumor tissues, which is expected to provide new targets for their treatment or prevention.

## Figures and Tables

**Table 1:** Primers used for PCR validation

Gene	Forward and Reverse primer
Lnc-PCAT1	F: 5' TGAGAAGAGAAATCTATTGGAACC3' R:5' GGTTTGTCTCCGCTGCTTTA 3'
miR-370-3p	F: 5' TCGGCAGGGCCTGCTGGGGTGG 3' R:5' CTCAACTGGTGTCGTGGA 3'
LRAT	F:5' TGATGCCCGACATCCTGTTG 3' R:5' ATGTTAGCTCCGTAGGCGAAG 3'
circ_0012586	F:5' GGAAAAGATCTACCCTGAGGAGC 3' R:5' GGGGGTGCCGAGTCAGATG 3'
miR-200b-5p	F:5' TCGGCAGGCATCTTACTGGGCA 3' R:5' CTCAACTGGTGTCGTGGA 3'
MFAP5	F:5' GCATCGGCCGGTTAAACAAT 3' R:5' TCACAGGGAGGAAGTCGGAA 3'
GAPDH	F:5' CATGAGAAGTATGACAACAGCCT 3' R:5' AGTCCTTCCACGATACCAAAGT 3'

**Table 2:** The designed sequences for lncRNA PCAT1 and circ_0012586 in the study

Name	Sequence
si-PCAT1-1	5'-CAAAGGAUAUAAGAUGCAUTT-3′
si- PCAT1-2	5'-CUGACGUCUUGCCAACUAATT-3′
si- PCAT1-3	5'-GAUGACGCAAAGGAACCUATT-3′
si-NC	5'-TTCTCCGAACGTGTCACGT-3'
si-circ_0012586-1	5'-TGACTTCCCGAGGTTCCGTCA-3'
si-circ_0012586-2	5'-CCTGACTTCCCGAGGTTCCGT-3'
si-circ_0012586-3	5'-TCCCGAGGTTCCGTCAGCCCT-3'

**Table 3:** Statistical Analysis of All of the Differently Expressed ncRNAs and mRNAs

DE RNAs	Total No.	No. of Down-regulated	No. of Up-regulated	The Most Down-regulated (Fold Change)	The Most Up-regulated (Fold Change)
mRNA	1219	626	593	AC132217.2 (-123.6)	CFAP47(42.5)
LncRNA	375	159	216	AC132217.1 (-7.68)	AC010197.1 (6.59)
miRNA	22	15	7	miR-483-3p (-6.65)	miR-375-3p (8.30)
CircRNA	667	345	323	circ_0020792(-6.91)	circ_0077054(12.47)

## Abbreviations

OS: osteosarcoma
ncRNA: non-coding RNA
ceRNA: Competing endogenous RNA
circRNA: circular RNAs
GO: Gene Ontology
KEGG: Kyoto Encyclopedia of Genes and Genomes
qRT-PCR: quantitative real-time PCR
MREs: microRNA response elements
SDS-PAGE: sodium dodecyl sulfate-polyacrylamide gel electrophoresis
PVDF: polyvinylidene difluoride
FC: fold change
FDR: false discovery rate
